# Correction: A selective fluorescent probe for an organophosphorous nerve agent mimic *via* an oxime-to-isoxazole cascade reaction

**DOI:** 10.1039/d6ra90009g

**Published:** 2026-02-04

**Authors:** Muhammad Shar Jhahan Khan, K. S. Al-Namshah, Muhammad Saleem, Ya-Wen Wang

**Affiliations:** a Department of Chemistry, Ghazi University Pakistan seemab35usa@gmail.com; b Chemistry Department, College of Science, Imam Mohammad Ibn Saud Islamic University (IMSIU) Riyadh 11623 Saudi Arabia KSNamshah@imamu.edu.sa; c Department of Chemistry, Ghazi University Pakistan msaleem@gudgk.edu.pk; d School of Chemistry, Southwest Jiaotong University China ywwang@swjtu.edu.cn

## Abstract

Correction for ‘A selective fluorescent probe for an organophosphorous nerve agent mimic *via* an oxime-to-isoxazole cascade reaction’ by Muhammad Shar Jhahan Khan *et al.*, *RSC Adv.*, 2026, **16**, 3720–3724, https://doi.org/10.1039/D5RA08944A.

The authors regret that one of the affiliations (affiliation *b*) was incorrectly shown in the original manuscript. The corrected list of affiliations is as shown herein.

The Royal Society of Chemistry apologises for these errors and any consequent inconvenience to authors and readers.

